# Acute cellular and vascular responses to photodynamic therapy using EGFR-targeted nanobody-photosensitizer conjugates studied with intravital optical imaging and magnetic resonance imaging

**DOI:** 10.7150/thno.37949

**Published:** 2020-01-20

**Authors:** Henriette S. de Bruijn, Vida Mashayekhi, Tom J.L. Schreurs, Pieter B.A.A. van Driel, Gustav J. Strijkers, Paul J. van Diest, Clemens W.G.M. Lowik, Ann L.B. Seynhaeve, Timo L.M. ten Hagen, Jeanine J. Prompers, Paul M.P. van Bergen en Henegouwen, Dominic J. Robinson, Sabrina Oliveira

**Affiliations:** 1 Center for Optical Diagnostics and Therapy, Dept. of Otolaryngology and Head & Neck Surgery, Erasmus MC Cancer Institute, Rotterdam, The Netherlands.; 2 Cell Biology Division, Dept. of Biology, Faculty of Science, Utrecht University, Utrecht, The Netherlands.; 3 Biomedical NMR, Biomedical Engineering, Eindhoven University of Technology, Eindhoven, The Netherlands.; 4 Division of Optical Molecular Imaging, Dept. of Radiology, Leiden University Medical Center, Leiden, The Netherlands.; 5 Amsterdam University Medical Centers, University of Amsterdam, Dept. of Biomedical Engineering and Physics, The Netherlands.; 6 Dept. of Pathology, University Medical Centre Utrecht, Utrecht, The Netherlands.; 7 Laboratory of Experimental Oncology, Dept. of Pathology, Erasmus MC, Rotterdam, The Netherlands.; 8 Pharmaceutics Division, Dept. of Pharmaceutical Sciences, Faculty of Science, Utrecht University, Utrecht, The Netherlands.

**Keywords:** Targeted, EGFR, Nanobody, Photosensitizer, Photodynamic therapy, Intravital microscopy.

## Abstract

Targeted photodynamic therapy (PDT) has the potential to selectively damage tumor tissue and to increase tumor vessel permeability. Here we characterize the tissue biodistribution of two EGFR-targeted nanobody-photosensitizer conjugates (NB-PS), the monovalent 7D12-PS and the biparatopic 7D12-9G8-PS. In addition, we report on the local and acute phototoxic effects triggered by illumination of these NB-PS which have previously shown to lead to extensive tumor damage.

**Methods:** Intravital microscopy and the skin-fold chamber model, containing OSC-19-luc2-cGFP tumors, were used to investigate: a) the fluorescence kinetics and distribution, b) the vascular response and c) the induction of necrosis after illumination at 1 or 24 h post administration of 7D12-PS and 7D12-9G8-PS. In addition, dynamic contrast enhanced magnetic resonance imaging (DCE-MRI) of a solid tumor model was used to investigate the microvascular status 2 h after 7D12-PS mediated PDT.

**Results:** Image analysis showed significant tumor colocalization for both NB-PS which was higher for 7D12-9G8-PS. Intravital imaging showed clear tumor cell membrane localization 1 and 2 h after administration of 7D12-9G8-PS, and fluorescence in or close to endothelial cells in normal tissue for both NB-PS. PDT lead to vasoconstriction and leakage of tumor and normal tissue vessels in the skin-fold chamber model. DCE-MRI confirmed the reduction of tumor perfusion after 7D12-PS mediated PDT. PDT induced extensive tumor necrosis and moderate normal tissue damage, which was similar for both NB-PS conjugates. This was significantly reduced when illumination was performed at 24 h compared to 1 h after administration.

**Discussion:** Although differences were observed in distribution of the two NB-PS conjugates, both led to similar necrosis. Clearly, the response to PDT using NB-PS conjugates is the result of a complex mixture of tumor cell responses and vascular effects, which is likely to be necessary for a maximally effective treatment.

## Introduction

Targeted strategies to deliver a photosensitizer to tumor cells have the potential to improve the therapeutic effect of photodynamic therapy (PDT) 1-5. PDT involves the administration of a photosensitizer and the application of light with the appropriate wavelength 1,2. After absorption of light by the photosensitizer, a range of photochemical reactions occur that, in the presence of oxygen, leads to the formation of predominantly singlet oxygen 1,2. The highly reactive singlet oxygen causes damage to nearby proteins and lipids resulting in cellular, vascular and immunological responses that ultimately encompass the PDT response 1. In general, clinically used photosensitizers are hydrophobic, which promotes cell binding but provides no tumor specificity. As a result, a 2 to 4 days interval between photosensitizer administration and light delivery is common to obtain an optimum tumor to normal tissue ratio. In addition, patients become light sensitive, for several weeks, due to skin-photosensitization. Increased tumor specific uptake of the photosensitizer has the potential to lead to significantly better tumor responses, much less normal tissue damage and a decreased skin photosensitization 1-3. Increased expression levels of specific receptors on tumor cells can be used to target these cells. In head and neck cancer, 83% of tumors show overexpression of the epidermal growth factor receptor (EGFR), which is commonly used as a target for various targeted therapies 6. For targeted PDT a range of approaches have been explored, such as the direct conjugation of photosensitizer to antibodies or peptides, or targeted drug delivery systems where multiple photosensitizer are loaded into a liposome or other nanoparticles 4,5,7-14.

We have been developing an alternative approach using nanobodies 15-18. Nanobodies are the smallest naturally derived antigen-binding fragments that consist of the variable domain of a heavy-chain antibody 19. The characteristics of nanobodies make them favorable for targeted drug delivery as they bind specifically and with high affinity to their antigens, are relatively small, stable, water soluble and have low immunogenic potential 19-22. The monovalent 7D12 and the biparatopic 7D12-9G8 nanobodies specifically target EGFR and compete with EGF for binding to EGFR 23. In contrast to the monovalent 7D12 nanobody, the biparatopic 7D12-9G8 leads to a more rapid EGFR internalization by inducing receptor clustering 15,24. After conjugation to the water soluble photosensitizer IRDye700DX, a silicon-phthalocyanine derivative that absorbs and emits NIR light, these nanobodies act as a carrier of photosensitizer and can be used for targeted PDT or tumor targeted fluorescence guided imaging 4,25,26.

Recently we have shown promising results *in vitro* and *in vivo* using nanobody-photosensitizer (NB-PS), as an alternative approach for targeted PDT 15,16. *In vitro* we have shown a clear relationship between level of EGFR expression, fluorescence intensity and PDT efficacy for both 7D12-PS and 7D12-9G8-PS 15. Subsequently, in an *in vivo* study employing an orthotopic tongue model transplanted with an oral squamous cell carcinoma expressing green fluorescent protein (OSC-19-luc2-cGFP), we used quantitative fluorescence spectroscopy to determine the NB-PS distribution in time after administration 16. The fluorescence intensity in tumor and normal skin tissue was significantly higher for 7D12-9G8-PS compared to 7D12-PS. 7D12-PS showed a peak fluorescence intensity in the tongue tumor already at 30 min after administration after which it slowly decreased. 7D12-9G8-PS showed a high fluorescence intensity in the tumor up to 4 h after administration after which it started to decrease. The tumor to normal ratio at 1 h after administration was 1.8±0.3 and 3.8±0.5, respectively. Although the tumor to normal ratio increased to 16.1±4.5 and 30.8±0.9, respectively at 24 h after administration, the tumor fluorescence intensity was significantly lower. Therefore, in that study, light was applied 1 h after administration for both NB-PS. Histological examination 24 h after PDT showed extensive tumor necrosis and damage to the vasculature in and close to the tumor 16.

These promising results encouraged us to further investigate, in the present study, the distribution of the NB-PS conjugates and PDT-induced response *in vivo* within tumor and normal tissue. The (sub-) cellular localization of photosensitizer is considered to be important as it determines the initial site of photodamage due to the short diffusion distance of singlet oxygen 27. Antitumor effects induced by PDT are known to be mediated not only by cellular damage but also by vascular responses 1. Interestingly, PDT, and more recently targeted PDT, have been shown to be capable of increasing the enhanced permeability and retention (EPR) effect by improving tumor vessel permeability 28-31. Since this could potentiate the delivery of other nanomedicines to the tumor site in future combined therapies, in this study we also carefully investigate the vascular effects of NB targeted PDT.

Intravital imaging in a skin-fold chamber model can be used to provide longitudinal information on the kinetics and localization of fluorophores in detail in a living animal, and be used to investigate direct effects on the vasculature 8,32-39. Therefore, in the present study, we used intravital imaging in the mouse skin-fold chamber model transplanted with the OSC-19-luc2-cGFP tumor, a tumor model we have previously investigated in the mouse tongue 16. We investigated the biodistribution of the conjugates and the vascular responses induced by NB-PS mediated PDT; constriction and leakage, as well as the induction of necrosis after illumination at 1 or 24 h post administration 16,40-43. To complement intravital microscopy, contrast-enhanced MRI and dynamic contrast enhanced (DCE) MRI were also used, as these have shown to be effective tools to determine the vascular effects and measure the microvascular status of tumors early after PDT 43-50. We employed the same tumor cell line but now grown subcutaneously in mice for DCE-MRI, to interrogate the microvascular status of the tumor and underlying muscle 50. Here, the degree of blood perfusion and vessel integrity can be quantified by measuring the tissue influx and wash out of a small gadolinium-based contrast agent. Histological examination of tissues collected directly after or up to 48 h after PDT was also used to determine the tumor necrotic fraction, vascular perfusion and damage to endothelial and normal tissue in both models.

## Materials and Methods

### Study design

Intravital microscopy and DCE-MRI were used to investigate the distribution and the direct effects of two EGFR-targeted NB-PS conjugates.

Intravital microscopy combined with the skin-fold chamber tumor model was used to study fluorescence distribution and PDT response longitudinally. Mice wearing the skin-fold chamber were divided over 6 groups (**Table 1**). Fluorescence distribution was investigated intravitally in groups 1-3 by imaging at 1, 2 and 24 h after administration of the NB-PS and before PDT. After the last time-point (24 h) PDT was performed in an attempt to use the high tumor to normal ratio (TNR) observed previously 16. In groups 4-6 PDT was performed 1 h after administration. Groups 3 and 6 were light only controls. PDT responses were investigated intravitally in all groups by imaging directly, 2, 24 and 48 h after PDT. At 2 h post illumination tetramethylrhodamine dextran was administered and imaged in all groups to investigate vascular leakage and flow. At 24 h post illumination the necrosis marker HQ4 41,42 was administered and imaged 24 h later. Tissue was harvested for *ex vivo* necrosis marker imaging and histological analysis.

DCE-MR imaging of the solid tumor model was used to investigate the PDT response in 2 groups. One group of animals (n=5) was injected with 7D12-PS and the control group (n=4) received saline. For practical reasons only one of the two NB conjugates could be investigated and 7D12-PS was chosen. To compensate for the prolonged gas-anesthesia necessary for the procedure, illumination was performed at 2 h instead of 1 h after administration. MR imaging was performed on 3 consecutive days, 1 day before illumination, approximately 1 h after illumination inside the bore of the scanner on the second day and 1 day after illumination, after which animals were sacrificed. Histological analysis was performed on a selection of 2 control animals and 3 7D12-PS mediated PDT treated animals, which received a perfusion marker (Hoechst 33342) 5 min prior to sacrifice.

### Nanobody conjugates

The nanobody-photosensitizer conjugates (NB-PS) 7D12-PS and 7D12-9G8-PS were prepared as described previously 15,16. His-tagged nanobodies were produced in E.coli BL21 and purified from the periplasmic fraction using Nickel-NTA agarose 23,51. The monovalent nanobody 7D12 binds to domain III of the EGFR, preventing EGF-binding to the receptor 52. The biparatopic nanobody 7D12-9G8 was composed of two nanobodies that bind to different epitopes on domain III and due to the short linker sequence they cannot bind simultaneously to the same receptor, therefore being able to create clusters of receptors 52. Conjugation of photosensitizer to the nanobodies was performed as described in Heukers *et al.* 2014 15, except that the molar ratios for conjugation was 1 to 4 for 7D12 and 1 to 3 for 7D12-9G8. In short, the nanobodies were incubated with the photosensitizer IRDye700DX (LI-COR Biosciences, Lincoln, Nebraska) for 2 h at room temperature. Afterwards, the NB-PS conjugates were separated from the free photosensitizer using three consequent Zeba spin desalting columns (Thermo Fisher Scientific, Perbio Science Nederland). The degree of the conjugation was determined using a Nanodrop spectrophotometer as recommended by the provider by measuring the absorbance at 280 nm and 689 nm. The purity of the NB-PS conjugates was determined on 15% SDS-PAGE and imaged on an Odyssey infrared scanner (LI-COR Biosciences) using 700 nm. Afterwards, PageBlue staining was performed to show the total protein content. The concentration of NB-PS administered was corrected for the degree of conjugation, so that every mouse received the same amount of photosensitizer, i.e. 6 nmol IRdye700DX).

### Cell line

The oral squamous cell carcinoma line, OSC-19-luc2-cGFP, was cultured in Dulbecco's Modified Eagle's Medium (containing 4.5 g D-Glucose/L, 110 mg Sodium Pyruvate/L) plus GlutaMAX^TM^, supplemented with 10% Fetal Bovine Serum, 1x Minimal Essential Medium non-essential amino acids solution and 1x Minimal Essential Medium vitamin solution as before 53.

### Intravital microscopy

#### Skin-fold chamber model

The mouse skin-fold chamber was prepared on the back of female Balb/c nu or BalB/cAnNRj mice and transplanted with the oral squamous cell carcinoma (OSC-19-luc2-cGFP) using a procedure adapted from previous studies 54-56. In brief, mice received analgesia 1 h (1 mg/kg rimadyl cattle s.c; Pfizer, Capelle a/d IJssel, NL) and anesthesia (75 mg/kg ketamine i.p.; Alfasan, Woerden, NL and 1 mg/kg medetomidine i.p.; Eurovet, Bladel, NL) 20 min before the procedure. The dorsal skin was folded and fixed between two frames after removal of one side of the skin in 1 cm diameter up to the fascia of the opposed skin. A tumor cell suspension of 5x10^4^ cells in 10 µL was injected superficially in the fascia/subcutaneous musculature and the window was closed with a sterile microscope cover glass of 12 mm diameter secured with a retaining ring. Glass spacers (thick cover glasses of 9 mm diameter) were placed on the epidermal side of the skin and another 12 mm circular microscopic cover glass was used to close the window on that side. Mice were housed individually in climate-controlled cabinets with an ambient temperature of 30 °C and a humidity of 70%.

Experiments started 6-9 days after preparation of the chambers. Mice were awake during all *i.v.* administrations. From two weeks prior to the experiments, all mice were fed a chlorophyll free diet (catalogue number 4208.00, Hope Farms b.v., Woerden, NL or #100208, Altromin, Germany) to minimize the contribution of pheophorbides to the autofluorescence emission spectrum. The animal ethics committee of the Erasmus MC approved the experimental protocols of the study.

#### Fluorescence distribution

The microscopic distribution of 7D12-PS and 7D12-9G8-PS was intravitally imaged under gas anesthesia at 1, 2 and 24 h after *i.v.* administration. Mice injected with physiological saline served as a control. A Zeiss Laser Scanner Confocal Microscope 510 equipped with 2x, 10x and 20x air Plan-Neofluar objectives, a heated stage and a gas anesthesia supply unit was used. Fluorescence images were recorded with the 2.5x (whole chamber), 10x (1 or 2 tumor areas of maximal 7.6 mm^2^ and 1 normal tissue area) and 20x objectives (3 z-stacks of 7 slices, 2 in tumor and 1 in normal tissue) using 633 nm excitation and long pass 650 nm detection for the nanobody-conjugates and 488 nm excitation and 505-530 nm band pass detection for the GFP signal of the tumor. Corresponding transmission images were recorded for orientation purposes using 488 nm light. Fluorescence and transmission images were recorded before, 1, 2 and 24 h after *i.v.* administration of the nanobody-conjugates under 2-3% isoflurane in oxygen anesthesia. Reference standards were recorded every day to correct for differences between experiments. Images were analyzed using ImageJ-Fiji. Regions of interest (ROI) were drawn around tumor and normal tissue areas excluding the large venules and arteries based on the transmission and GFP images. The ROI's for normal tissue were drawn in regions without any GFP signal and distant from larger blood vessels. Integrated density was determined and normalized to an area of 5000 µm^2^ rather than the mean fluorescence intensity of the pixels since the fluorescence was expected to be heterogeneous due to the receptor specific binding of the nanobody conjugates and the spatial resolution of the images. The integrated density was corrected for the dark current, variation in the reference standard and the individual autofluorescence. For each animal, time point and tissue type, 2-6 regions of interest's were drawn and averaged. Colocalization analysis between the GFP and red fluorescence signals was performed after correction for the dark current, using the Coloc2 plugin.

In parallel, for *ex vivo* microscopy, skin-fold chamber tissue of 4 mice was dissected at 1 or 24 h after administration of 7D12-PS or 7D12-9G8-PS (group 1, 2, 4 and 5). Tissue was snap-frozen and stored at -80 °C. Cryosections were made and stained with anti-CD31 Brilliant Violet (AntibodyChain BV, The Netherlands) for fluorescence microscopy. Images were collected using a Leica SP5 Microscope equipped with 40x oil Plan-Neofluar objective, 633 nm excitation and 650-800 nm HyD detection for the NB-conjugates and 405 nm excitation and 420-450 nm detection for the Brilliant Violet signal. Reference standards were recorded to correct for differences between experiments.

#### PDT

Illumination was performed under gas anesthesia on the microscope stage 1 or 24 h after *i.v.* administration of 7D12-PS or 7D12-9G8-PS. A 690 nm laser (ML7700, Modulight, Inc. Tampere, Finland) and a frontal light distributor (Medlight SA, Ecublens, Switzerland) was used to deliver a dose of 100 J.cm^-2^ at an irradiance of 50 mW.cm^-2^. Mice injected with physiological saline served as a light only control.

#### Cellular response to PDT

The GFP signal of the tumor cells was used to observe changes in the morphology in time after PDT. Necrosis was detected intravitally using the necrosis avid agent HQ4 (20 nMol/mouse 41,42) *i.v.* administered 24 h post PDT and imaged 24 h later using 633 nm excitation and 650 nm long pass detection. Tissue was dissected for *ex vivo* necrosis avid agent imaging and histological analysis. The skin-fold chamber tissue was cut in two halves through the tumor, snap-frozen in liquid nitrogen and stored in -80 °C until sectioning. Sections were collected at three depths with alternating thickness of 50 and 8 µm for HQ4 fluorescence imaging and histology respectively and stored in -80 °C until imaging. The 50 µm sections were defrosted, dried and imaged unfixed and uncovered using a Zeiss laser scanner confocal microscope. Fluorescence images were recorded with the 10x objective using 633 nm excitation and long pass 650 nm detection for HQ4 fluorescence and 488 nm excitation and 505-530 nm band pass detection for the GFP signal of the tumor. Corresponding transmission images were recorded for orientation purposes using 488 nm. Images were analyzed using ImageJ-Fiji. Regions of interest were drawn around tumor and normal tissue areas. The ROI's selection of tumor were created by thresholding the GFP signal and creating a mask of it to ensure that only tumor tissue was measured. The fluorescence intensity was corrected for the dark current and variation in the reference standard for the different recording days.

Thereafter tissues were formalin fixed and paraffin embedded (FFPE) for histological examination. The FFPE tissues were sectioned and stained with hematoxylin and eosin (H&E) and analyzed as described by van Driel *et al.* 16.

#### Vascular response to PDT

The vascular responses to PDT were investigated using both transmission imaging and tetramethylrhodamine dextran imaging. Tetramethylrhodamine dextran (2000 kDa, 1 mg/ml, 0.1 mg mouse, D7139, Thermo Fisher Scientific, Molecular Probes, Eugene, Oregon, USA) was administered 2 h post PDT and imaged intravitally within 20 min using 543 nm excitation and 560-615 nm bandpass detection and the 20x objective. The number of animals showing leakage in tumor or normal tissue, *i.e.*, tetramethylrhodamine fluorescence outside the vessels, was counted. Tumor vascular flow was scored based on the appearance of the tetramethylrhodamine fluorescence according to three criteria, *i.e.*, lack of flow (no fluorescence in the vessels), reduced flow (disrupted fluorescence in the vessels) or normal flow (fluorescence confined to the vessels). The proportional area of tumor tissue that was affected by each criterion was estimated. Transmission images using 488 nm were recorded immediately, 2, 24 and 48 h after PDT using the 2.5x objective to investigate changes in vascular architecture. The severity of the changes in vascular architecture in normal tissue was scored as follows: No change in vascular lumen and flow visible (-), minimal changes in vascular lumen of the larger vessels and/or capillary shutdown (+), severe changes in vascular lumen and flow visible in areas (++) and severe changes in vascular lumen and no flow visible (+++).

### DCE-MRI

#### Solid tumor model

Female BALB/c nude mice (Charles River) aged 8-11 weeks were subcutaneously injected with 1x10^6^ cells in the right hind limb. Tumors became palpable after 3 to 6 days and were measured with a caliper every 2 or 3 days. MRI experiments were started when the tumors reached a volume of approximately 300 mm^3^. The Animal Care and Use Committee of Maastricht University approved the protocol. All applicable institutional and/or national guidelines for the care and use of animals were followed.

#### PDT

Mice were anesthetized during injection of 7D12-PS or physiological saline until PDT and MRI imaging using 2-3% isoflurane in oxygen anesthesia. Body temperature was monitored with a rectal probe and maintained at 37 °C using a warm water circuit. Respiration rate was monitored using a pressure balloon. PDT was performed 2 h after administration and not 1 h to compensate for the lower metabolism caused by the prolonged gas anesthesia needed for the procedures. Light was delivered using a 690 nm laser (ML7700, Modulight, Inc. Tampere, Finland) via a fiber and collimating lens into the MR magnet to a dose of 100 J.cm^-2^ at an irradiance of 50 mW.cm^-2^. A black paper mask was placed surrounding the tumor to protect the rest of the animal from stray light.

#### MRI

Experiments were performed using a 7T MRI scanner (BioSpec 30/70 USR, Bruker, Billerica, Massachusetts, USA) with a 72 mm diameter quadrature transmit and receive birdcage coil. Briefly, *T_2_*-weighted anatomical reference scans were acquired followed by a 3D pre-contrast *T_1_* map. Then, a 15 min 3D DCE-MRI scan was performed with a temporal resolution of 3.5 s, to capture the dynamic influx of contrast agent (Dotarem) in the tissue. Based on the DCE-MRI data, all pixels were classified as enhanced or non-enhanced, and the following variables were calculated for each pixel: the area under the curve (AUC) of the Dotarem concentration, and *K^trans^* (transfer constant describing contrast exchange between blood plasma and the extravascular extracellular space). For anatomical reference a *T_2_* weighted multi-slice spin echo scan was acquired with 12 to 14 axial slices, covering the entire tumor. Scan parameters were: TR=1000ms, TE=30 ms, matrix=128x128, slice thickness=1.0 mm, slice gap=0.1 mm, FOV=4x4 cm^2^. For *T_1_*-mapping, a 3D FLASH sequence with variable flip angle was used 57. Sequence parameters were: TR-20 ms, TE=3.2 ms, 7 flip angles (2º, 3º, 5º, 7º, 10º, 13º and 20º), matrix=128x128x39, FOV=40x40x22 mm^3^. DCE-MRI was performed using the same 3D FLASH sequence with shorter TE and TR and a fixed flip angle (1 ms, 3 ms and 7º respectively) and an acquisition matrix of 128x128x17 (zero-filled to 128x128x39). Two min after the start of the scan, a dose of 0.3 mmol/kg b.w. Dotarem (Guerbet, Villepinte, France) was injected in 5 s using a syringe pump (Fusion 100, Chemyx Inc., Stafford, 153 TX, USA), followed by a saline flush.

Manual segmentation of tumors was performed in ITK-SNAP 58, based on *T_2_*-weighted spin echo images. Tumor was hyper intense compared to muscle tissue. All further analysis was performed using home-made scripts in Matlab 2016a (Mathworks, Natick, MA). Muscle ROI's of 5x5 pixels were selected in four central tumor slices, with 1 pixel spacing to the tumor border and centered on the optical axis of the light beam. *T_1_* maps were reconstructed pixel by pixel by fitting the data signal intensities at different FA to the spoil gradient echo sequence equation: 
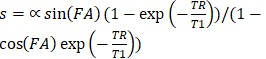
. Dynamic changes in *R_1_(t) = 1/T_1_(t)* were calculated from the DCE-MRI scan, based on the pre-contrast relaxation rate *R_1,pre_ = 1/T_1,pre_* and the signal during contrast agent influx, using the same equation. Next, Dotarem concentration curves *C(t)* were calculated using *R_1_(t)=R_1,pre_ + r_1_C(t)*, where *r_1_*= 3.53 s^-1^mM^-1^ is the longitudinal relaxivity of Dotarem 59. Pixels were classified as non-enhanced when the median of the concentration curve after time of contrast injection was smaller than twice the SD before injection. Tracer kinetic modelling was performed using the standard Tofts-Kermode model 60, to estimate *K^trans^* and *v_e_*(the volume fraction of the extravascular extracellular space). The arterial input function was defined as a bi-exponential function with amplitudes *A_1_* = 5.36 mM and *A_2_* = 1.27 mM, and time constants 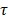
_1_ =5.36 s and 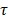
_2_ = 915 s, as previously described 49.

#### Histological analysis

The subcutaneous solid tumor tissue samples were collected 5 min after administration of Hoechst 33342 (*i.v.,* 32 mg/kg b.w. B2261, Sigma Aldrich, St. Louis, MO, USA), snap-frozen in liquid nitrogen and stored in -80 °C until sectioning and analysis. A series of sections were collected from a central and peripheral transverse plane and subjected to either hematoxylin and eosin staining (H&E) or CD31 immunofluorescence staining to detect endothelial cells. For CD31 detection, sections were fixed in acetone (5 min RT), air dried, incubated with biotin-conjugated rat anti-mouse CD31 antibodies (1 h 1:250 RT, #102503, Biolegend, San Diego, CA, USA) and DyLight 649-conjugated streptavidin (1 h 1:100 RT, #405224, Biolegend). Bright field and fluorescence imaging were performed by mosaic acquisition at 40x and 20x respectively. Viable and necrotic regions in H&E were manually segmented in ImageJ 1.51. All other data analysis, including segmentation of tumors in CD31 images were performed using home-made scripts in Matlab 2016a. In the CD31 images, tissue was classified as perfused or non-perfused by applying a manually selected threshold on the fluorescence signal intensity of Hoechst 33342.

### Statistics

Results of the fluorescence intensity of NB-PS or necrosis marker are presented as weighted mean ± SD, weighted by the SD of the mean fluorescence intensity of 2-6 ROI's at a certain time point for a certain tissue type. For each tissue type and time point the individual fluorescence intensity at a certain time point was weighted by its SD to result in a weighted mean calculated by averaging the different ROIs and weighted by the SD of that mean. The significance of differences was determined using the student t-test/ANOVA/SNK and p<0.05 was considered significant. The Spearman's rank correlation coefficient of the colocalization of the photosensitizer with the GFP fluorescence is presented as mean ± SEM and statistically analyzed using the student t-test and p<0.05 was considered significant.

## Results

### Fluorescence distribution of NB-PS conjugates

**Figure 1:**
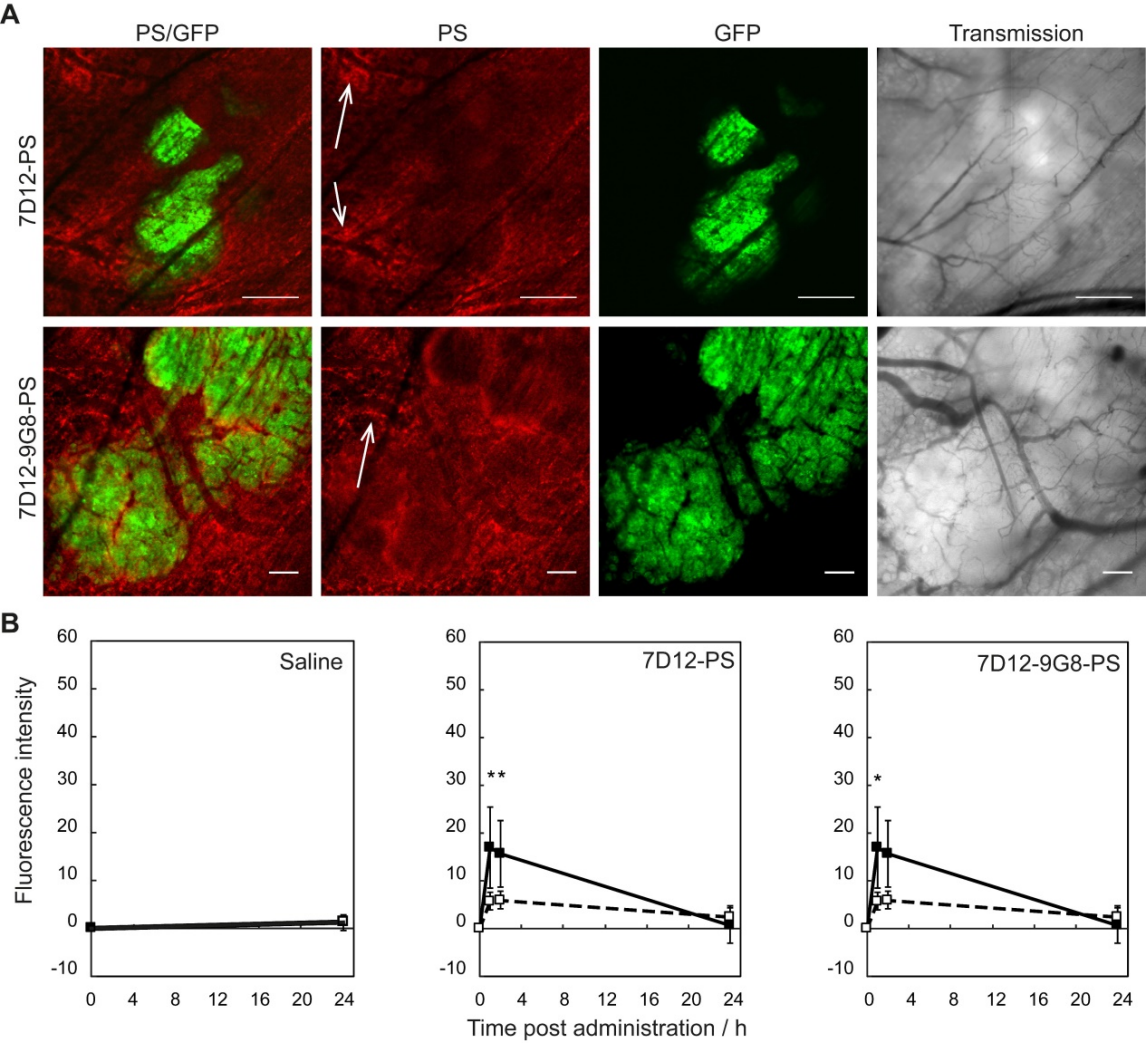
(A) Example of intravital fluorescence images recorded of the tumor in the skin-fold chamber 1 h after administration of 7D12-PS or 7D12-9G8-PS. Bar is 200 µm. White arrows highlight fluorescence close to vessels that surround tumor tissue. (B) Fluorescence intensity in tumor (solid squares and lines) and normal tissue far from tumor and not showing GFP signal (open squares and dashed lines) in the skin-fold chamber after administration of physiological saline, 7D12-PS or 7D12-9G8-PS. Weighted mean ± SD, n=3, 6, 8 respectively. Significant differences between tumor and normal tissue with p<0.05 (*).

Intravital microscopy imaging was performed to determine potential differences in the distribution between the two EGFR-targeted NB-PS in tumor and in normal tissue immediately surrounding the tumor (Figure 1A). With respect to the intensity, both conjugates showed more fluorescence in tumor than in distant normal tissue, at either 1 or 2 h after administration with p=0.04 and 0.03 for 7D12-PS, and p=0.04 and 0.06 for 7D12-9G8-PS, respectively (Figure 1B). The tumor to normal tissue ratio for the two conjugates was significantly higher for 7D12-9G8-PS (p<0.01) but only at 2 h after administration. The tumor to normal tissue ratio at 1 and 2 h after administration was 3.0±0.7 and 2.6±0.4 for 7D12-PS, and 2.9±0.6 and 4.0±0.2 for 7D12-9G8-PS, respectively. Comparing the two conjugates, more fluorescence was observed in tumor and normal tissue after 7D12-9G8-PS administration at all time points.

**Figure 2:**
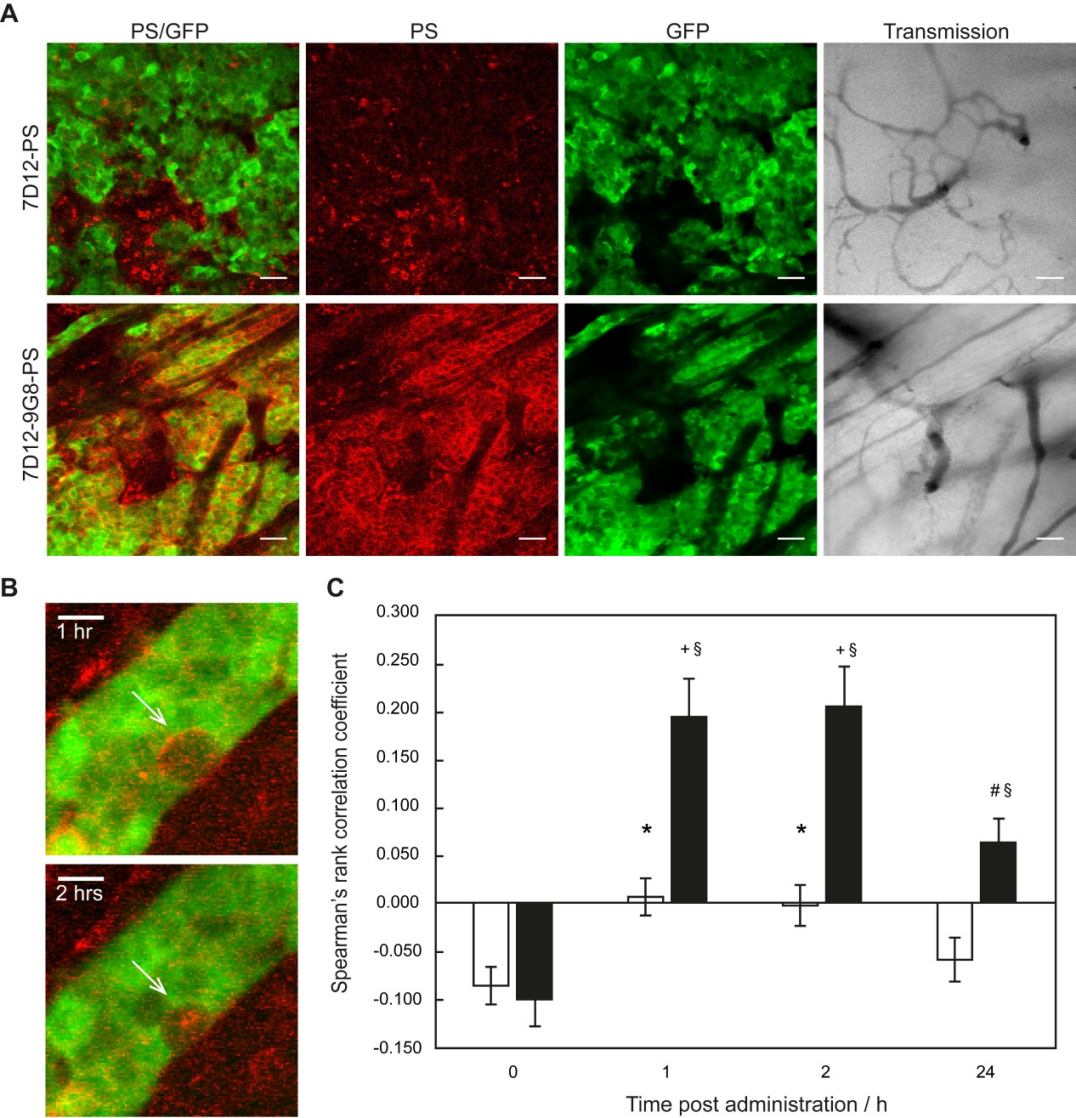
(A) Example of high magnification fluorescence images recorded 1 h after administration of 7D12-PS and 7D12-9G8-PS. Bar is 50 µm. (B) Consecutive images recorded in one animal at 1 and 2 h after administration of 7D12-9G8-PS fluorescence (red) in tumor (green) cells. Bar is 20 µm. (C) The Spearman's rank correlation coefficient for GFP fluorescence of tumor (green) and NIR fluorescence (red) of 7D12-PS (white bars) and 7D12-9G8-PS (black bars). Mean ± SEM and n=6 and 7 respectively. Significantly difference between time before and after administration with p<0.01 (*), p<2x10^-4^ (#) or p<2x10^-6^ (+). Significant difference between 7D12-PS and 7D12-9G8-PS with p<0.003 (§).

Distinct differences could be observed in the sub-cellular distribution of the two NB-PS conjugates using z-stack imaging with a higher magnification (Figure 2A). 7D12-9G8-PS showed an intense, membrane localized, fluorescence pattern in tumor cells. This was different after 7D12-PS administration, for which the fluorescence in the tumor tissue was more diffuse. In one animal we were able to image the internalization of 7D12-9G8-PS (Figure 2B). At 1 h after administration the fluorescence was visible at the membrane, and at 2 h fluorescence was clearly visible in the interior of the cell.

Colocalization analysis between the NB-PS fluorescence and the GFP signal in the tumor showed significant higher Spearman's rank correlation coefficients for 7D12-PS at 1 and 2 h after administration, compared to before administration, suggesting tumor specific binding with p<0.01 for both time points (Figure 2C). For 7D12-9G8-PS, all time points after administration showed significant higher Spearman's rank coefficients compared to before administration with p<2x10^-6^ for 1 and 2 h, and p<2x10^-4^ for 24 h after administration. Overall, 7D12-9G8-PS showed a significantly higher correlation between NB-PS fluorescence and GFP signal compared to 7D12-PS at all time points with p<0.003.

**Figure 3:**
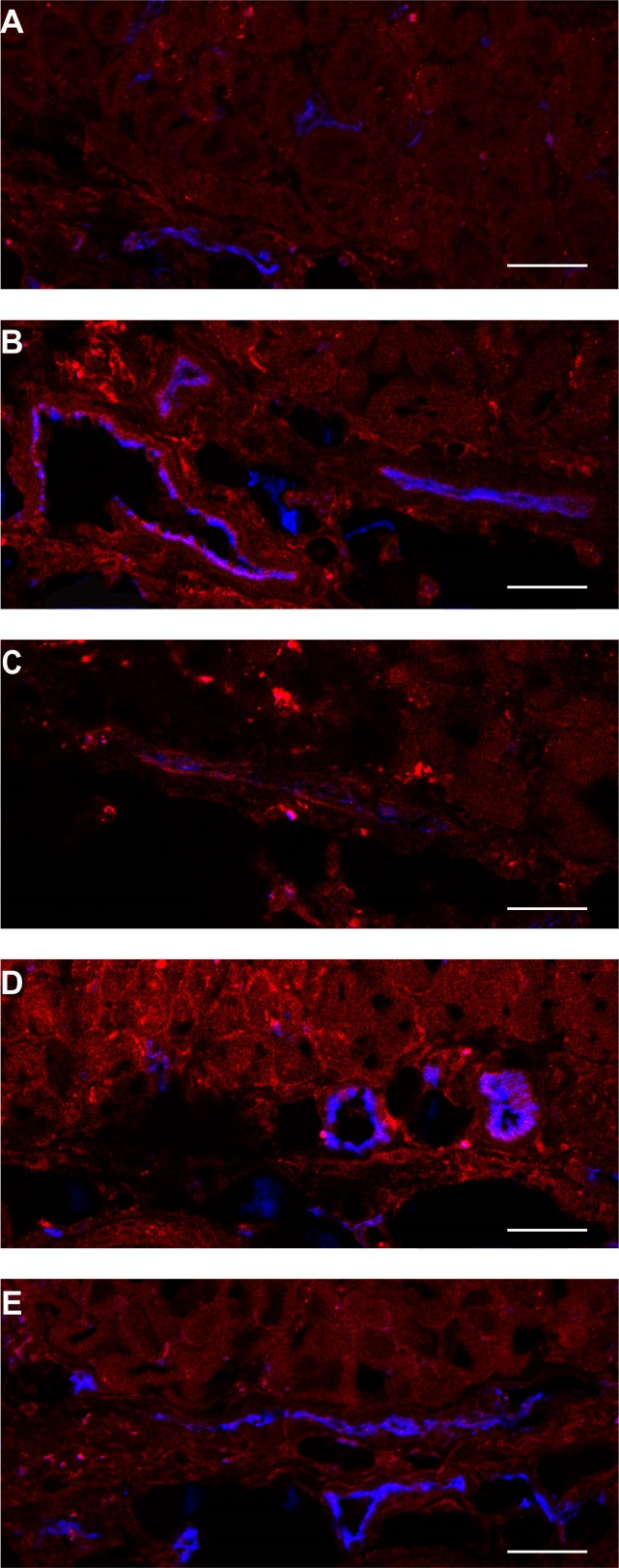
Example of PS fluorescence in the subcutaneous musculature and lower dermis of the skin-fold chamber tissue after administration of (A) saline, 7D12-PS at (B) 1 h or (C) 24 h after administration, or 7D12-9G8-PS at (D) 1 h or (E) 24 h after administration. Photosensitizer fluorescence is depicted in red, CD31 in blue and colocalization is shown in magenta. Bar is 50 µm.

Normal tissue showed a heterogeneous fluorescence pattern. For both conjugates, fluorescence was present in close proximity of vasculature in chambers, with and without tumor (white arrows in Figure 1A). Line profiles obtained perpendicularly over larger vessels showed up to a 4x higher fluorescence intensity close to the vessels compared to further from the vessel (data not shown). In order to determine the localization of the fluorescence more carefully, tissue was harvested at 1 or 24 h after administration of the NB-PS, sectioned and stained for endothelial cells (CD31 fluorescent detection). *Ex vivo* fluorescence microscopy showed high fluorescence intensities for both NB-PS in normal tissue associated with the endothelial cells of (larger) blood vessels (Figure 3).

### Responses to nanobody targeted PDT

Since the acute response to PDT is a complex mixture of cellular and vascular responses, we investigated both using different techniques. Initial tumor cell responses were assessed by observing changes in the GFP signal using intravital imaging. Vascular responses were investigated with intravital fluorescence imaging for vascular flow and leakage (tetramethylrhodamine dextran), intravital transmission imaging for vascular architecture; and DCE-MRI for assessment of vascular perfusion. These were combined with *ex vivo* histology for evaluation of necrosis marker (HQ4) and of (permanent) tissue necrosis.

#### Tumor cellular responses

**Figure 4:**
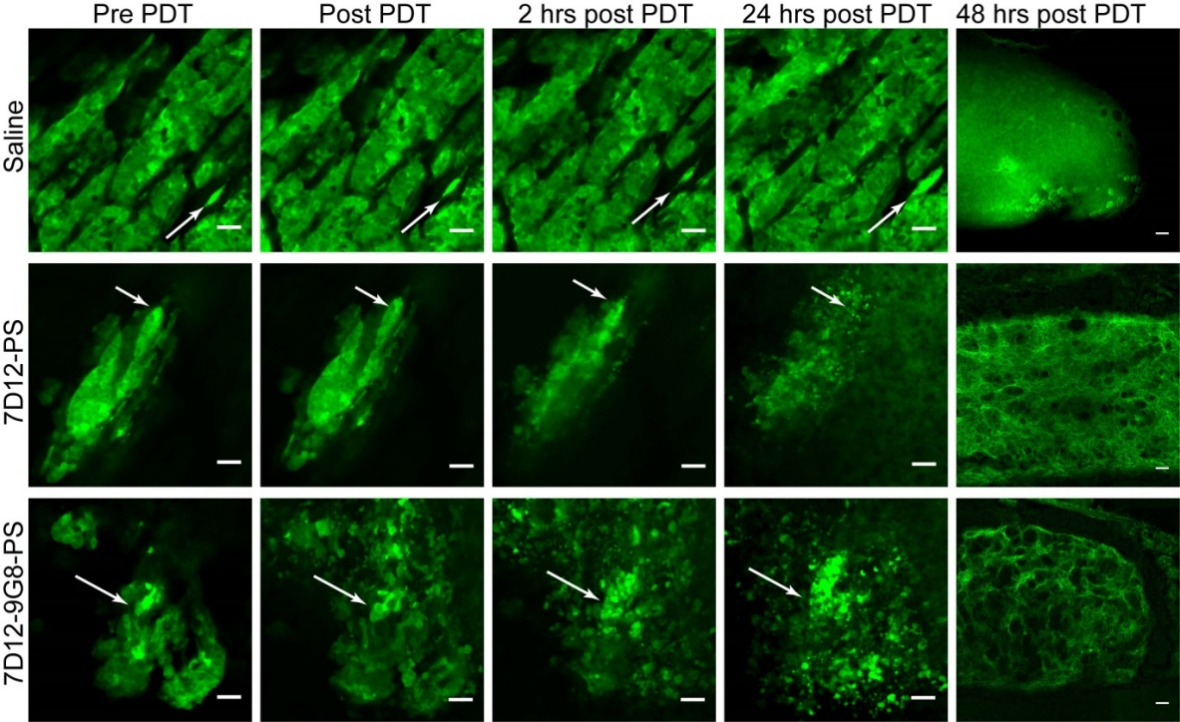
Examples of the effect of PDT on the morphology of tumor cells intravitally imaged in the skin-fold chamber, at 1 h post administration of saline (top), 7D12-PS (middle) and 7D12-9G8-PS (bottom) up to 24 h after PDT. Arrows point to the same cell cluster in every image for each group. The last column displays the GFP fluorescence images of cryosections obtained 48 h after PDT. Bar 50 µm.

Distinct changes to the morphology of tumor cells were observed in the GFP fluorescence images recorded in time, after illumination at 1 h (Figure 4). Before illumination, the GFP signal was confined to the tumor cells, with high fluorescence intensity in the cytoplasm compared to the nucleus. In light only controls, the number of GFP containing cells increased in the 24 h after illumination, thus reflecting tumor growth. In NB-PS treated animals that were illuminated 1 h after administration, the normal cellular GFP fluorescence pattern, in some cases, changed significantly. In those cases the GFP fluorescence appeared more diffuse and/or confined to circular spots of different diameters with high fluorescence intensities, suggesting tumor cell damage. This was seen in more animals treated with 7D12-9G8-PS than with 7D12-PS (10/13 fields vs 3/12 fields, respectively) and was not observed for PDT treatments 24 h after administration (data not shown). Fluorescence imaging of cryosections of the tissues collected 48 h after PDT showed similar results (Figure 4).

#### Vascular responses

**Figure 5:**
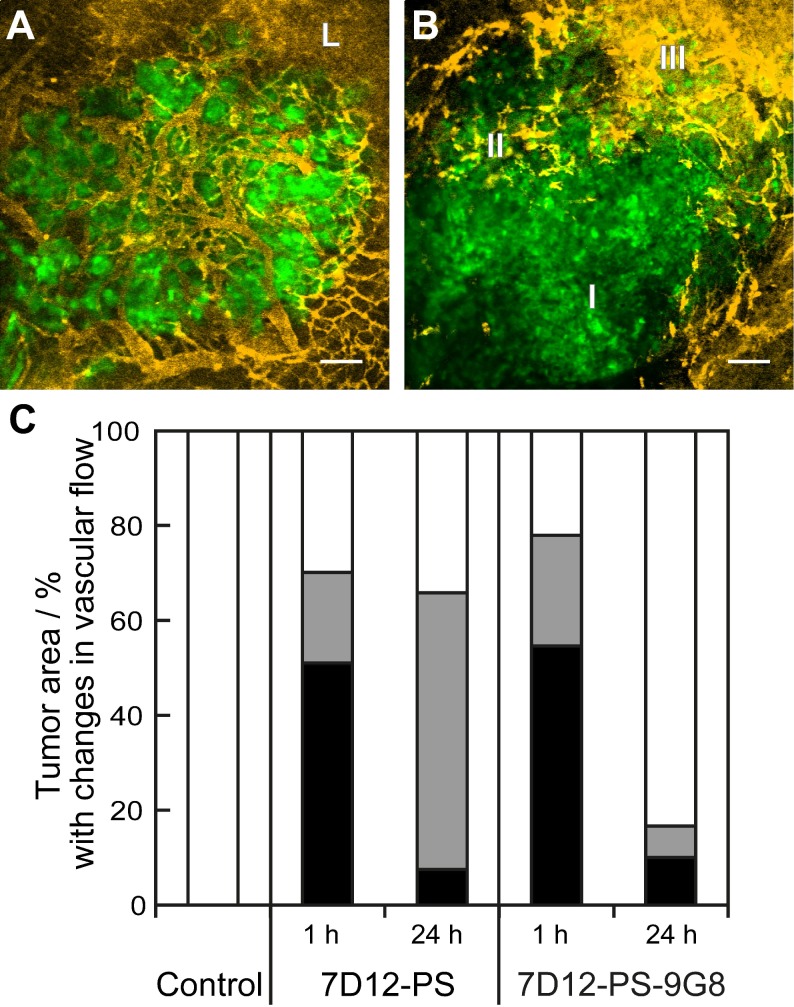
Representative images showing the different tumor vascular responses observed 2 h after PDT recorded using GFP fluorescence (green) and tertramethylrhodamine dextran fluorescence (yellow). Bar is 100 µm. (A) Normal blood flow in the tumor and leakage (L) just outside the tumor. (B) Tumor with an area of no flow (I), reduced flow (II) and normal flow with leakage (III). (C) Qualitative analysis of vascular flow in tumor determined 2 h after PDT using 7D12-PS or 7D12-9G8-PS and illuminated either at 1 or 24 h post administration. The relative area in tumor that showed either no flow (black), reduced flow (gray) or normal flow (white) was determined and averaged in 6 animals.

Vascular leakage of tumor and normal tissue vessels was observed for both NB-PS treated groups. But there was no clear relationship between the number of tumors that showed leakage and the conjugate used or the time at which the light was delivered (Table 2). The proportional area of tumor that showed either lack of flow or reduced flow 2 h post PDT was similar for both conjugates when light was applied 1 h after administration (Figure 5). The severity of the vascular response decreased significantly when the light was delivered at 24 h after administration, as the size of the area with lack of flow decreased from 51% to 8% with p=0.029 for 7D12-PS and from 55% to 10% with p=0.033 for 7D12-9G8-PS. Comparing the two conjugates, a larger area of tumor showed reduced flow 2 h post illumination at 24 h after administration of 7D12-PS compared to 7D12-9G8-PS (Figure 5).

**Figure 6:**
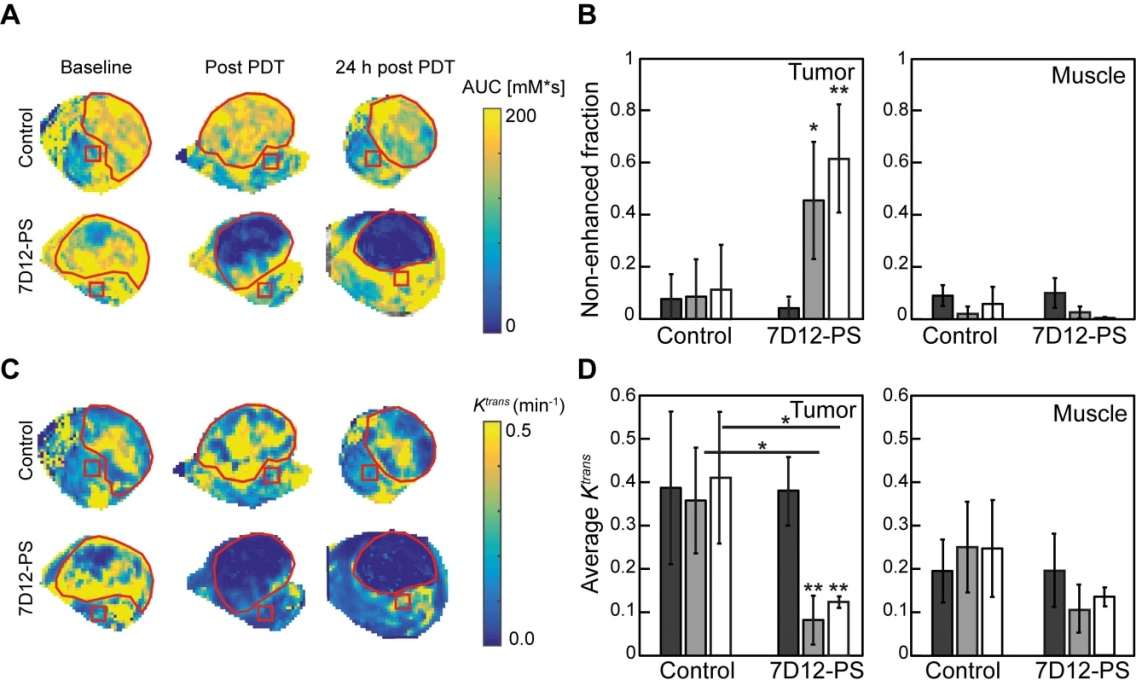
(A) Representative AUC maps of a control and 7D12-PS mediated PDT treated animals recorded at baseline, post PDT and 24 h post PDT. The red contours outline the tumor, the red squares represent muscle ROI. (B) Group averages of the non-enhanced fraction pre PDT (black), post PDT (grey) and 24 h post PDT (white) in enhanced tumor and muscle ROI's. Significant differences from pre PDT in same group are indicated with p=0.05 (*) or p=0.005 (**). (C) Representative *K^trans^* maps of a control and 7D12-PS mediated PDT treated animal recorded at baseline, post PDT and 24 h post PDT. The red contours outline the tumor, the red squares represent muscle ROI. (D) Group averages of the mean *K^trans^* pre PDT (black), post PDT (gray) and 24 h post PDT (white) in enhanced tumor pixels and muscle ROI's. Significant differences from pre PDT from same group or between groups are indicated with p=0.05 (*) or p=0.005 (**).

DCE-MRI imaging of the solid tumors after 7D12-PS mediated PDT showed tumor non-enhancement values that were in agreement with the proportion of tumor showing lack of flow in the skin-fold chambers (Figure 6A-B). Directly after and 24 h after 7D12-PS mediated PDT, 42.8±22.6% and 61.9±21.4% of the tumor showed non-enhancement, *i.e.*, loss of contrast agent uptake, which was significantly increased compared to baseline (p=0.01 and 4.6810^-4^, respectively). In agreement with the loss of contrast agent, uptake in tumors showed negligible *K^trans^*values throughout the whole tumor at both time points (Figure 6C-D). The remaining pixels with residual contrast enhancement mostly showed lower *K^trans^*values compared to untreated tumor tissue. Most parts of the underlying muscle showed a decrease in *K^trans^*immediately and 24 h after PDT, suggesting impaired microvascular perfusion, although this was not statistically significant (p=0.13 and 0.24 respectively).

**Figure 7:**
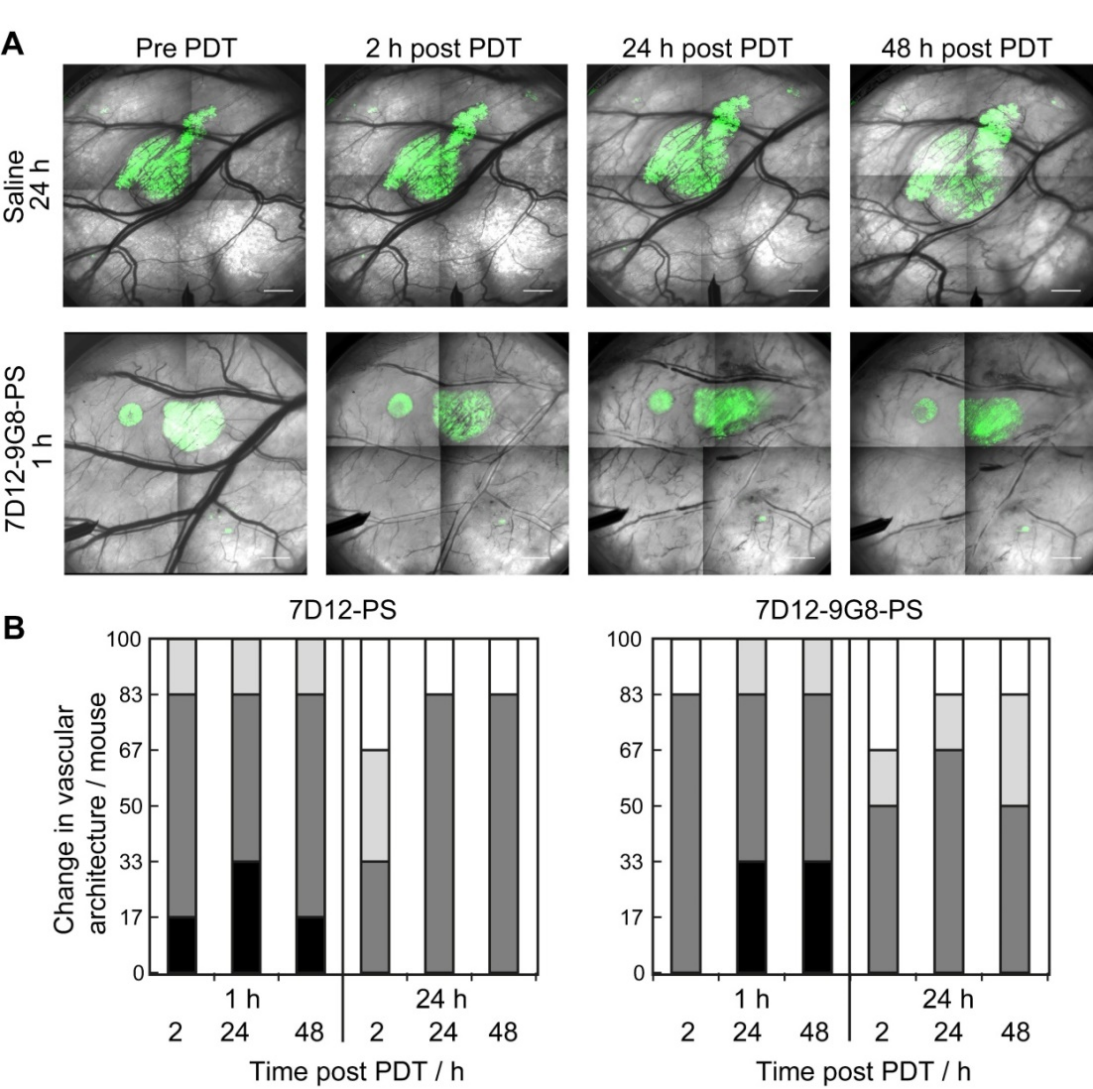
(A) Representative images of the changes in vascular architecture in the skin-fold chamber tissue collected longitudinally before and in time after NB-PS mediated PDT delivered 1h post administration. Bar = 1000 µm. (B) Severity of the change in vascular architecture in normal tissue per mouse scored at different time points after PDT at 1 or 24 h using 7D12-PS or 7D12-9G8-PS. No change in vascular lumen and normal blood flow was scored white (-), capillary shutdown and or small changes in vascular lumen (light grey, +), severe changes is vascular lumen but blood flow observed (dark grey, ++) and severe changes in vascular lumen and no flow observed (black, +++).

The normal skin vasculature showed constriction of the larger arterioles and venules, and also changed vascular architecture post NB-PS mediated PDT. Comparing the two conjugates, slight differences were observed, 1 animal showed more severe constriction 2 h post PDT using 7D12-PS compared to 7D12-9G8-PS but this was reversed 48 h after PDT. Illumination at 24 h after administration resulted in slightly more vascular constriction at 24 and 48 h post PDT for 7D12-PS compared to 7D12-9G8-PS. Overall, these effects were not significantly different for the two conjugates, only more severe for illumination at 1 h compared to 24 h after administration (Figure 7).

#### Tumor and normal tissue viability and necrosis

**Figure 8:**
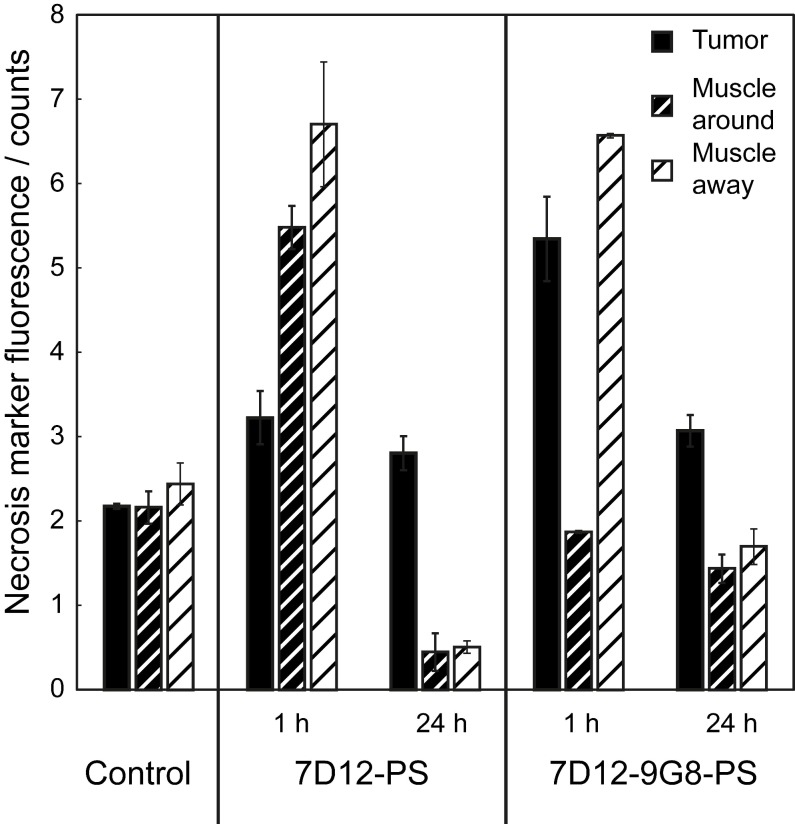
Fluorescence intensity of the necrosis marker HQ4 in cryosections of the skin-fold chamber collected 48 h after PDT at 1 h post administration of physiological saline, 7D12-PS, 7D12-9G8-PS, and after PDT at 24 h post administration of 7D12-PS and 7D12-9G8-PS ROI's were drawn around tumor (black bar) and sub cutaneous musculature around and away from tumor (fat or thin dashed bar) respectively. Weighted mean ± SD, n=1-6 animals per group and 2-3 sections per animal.

The PDT induced vascular responses and edema significantly changed the tissue optical properties of tissues in the window chamber, rendering intravital imaging of the necrosis avid agent HQ4 48 h after PDT impossible. Therefore, imaging of HQ4 was performed on cryosections of excised tissues. More fluorescence of HQ4 was observed in tumors treated with NB targeted PDT compared to the light only controls (Figure 8). While it is unknown if there is a linear relationship between HQ4 fluorescence intensity and necrosis, the data suggests more necrosis for illumination at 1 h after administration compared to 24 h after administration.

**Figure 9:**
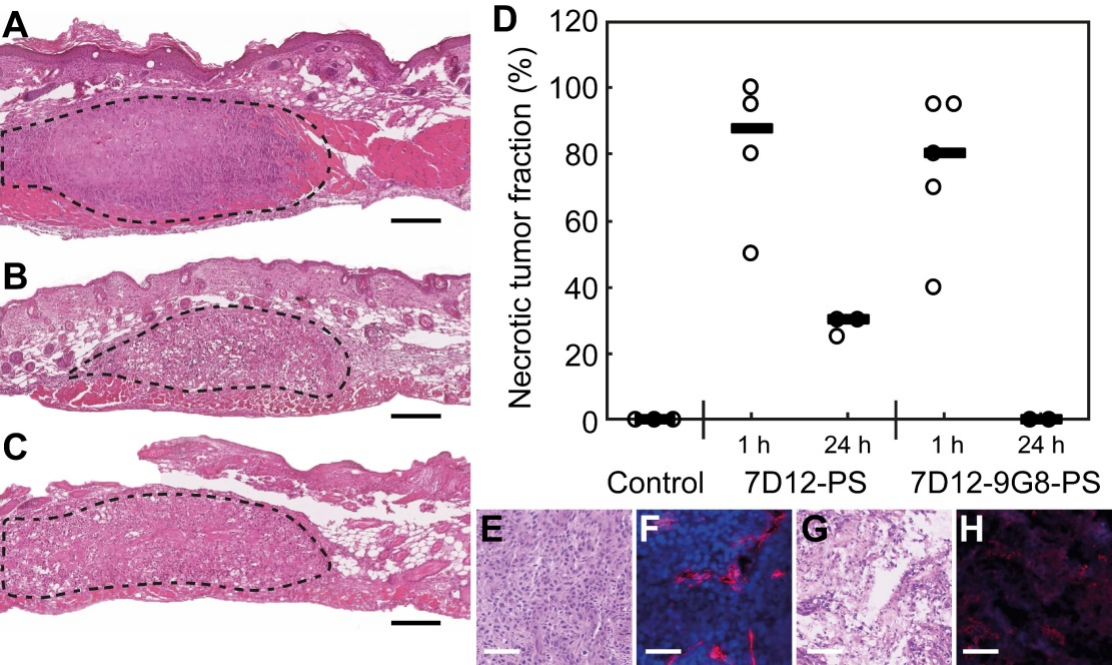
Example of H&E stained sections of the skin-fold chamber 48 h after PDT at 1 h post administration of (A) physiological saline, (B) 7D12-PS or (C) 7D12-9G8-PS. Tumor is outlined with dashed lines. Black bar is 200 µm. (D) Individual percentages of tumor necrosis and the median per treatment. Representative examples of (E,G) H&E bright-field images and (F,H) CD31 & Hoechst 33342 fluorescence images of (E,F) control and (G,H) 7D12-PS mediated PDT treated solid tumors. CD31 is displayed in red and Hoechst in blue. White bar is 100 µm.

H&E staining of the skin-fold chamber tissues also showed more tumor necrosis for illumination at 1 h compared to 24 h post administration (Figure 9A-D). PDT at 1 h after administration induced 88 or 80% necrosis in tumors compared to 30 or 0% at 24 h for 7D12-PS and 7D12-9G8-PS, respectively. Complementary to this, a similar average necrotic tumor fraction was determined in H&E sections of the solid tumor model for treatment with 7D12-PS mediated PDT at 2 h (89±9% , compared to 23±20% in light only controls). Fluorescence imaging of Hoechst 33342 in adjacent sections showed co-localization of those areas with the non-perfused areas (74±17% and 23±33%, respectively). Tumors treated with 7D12-PS mediated PDT hardly showed any uptake of Hoechst 33342 and fewer blood vessels were detected. Blood vessels had weak staining and indistinct morphology, possibly due to disruption and closed lumina (Figure 9E-H).

Concerning normal tissues, damage to these in the skin-fold chamber model was similar for both NB-PS conjugates used (Table 3). PDT delivered 1 h after administration resulted in severe damage to the normal tissues close to tumor. Away from the tumor, the damage was mild. No damage to normal tissues was observed when PDT was performed 24 h after administration. Also, the skin-fold chambers without tumor showed no damage to normal tissues 48 h after PDT at either 1 or 24 h post administration (n=1 per group).

## Discussion

The present study was designed to investigate the biodistribution of two NB-PS, the cellular responses to and the vascular effects of EGFR-targeted PDT *in vivo* using intravital microscopy and DCE-MRI, complemented with *ex vivo* analysis of necrosis.

The overall fluorescence kinetics determined with intravital microscopy were similar to those we have previously reported for the orthotopic mouse tongue model using quantitative fluorescence fiber optic spectroscopy 16. In both cases, we have shown more NB-PS fluorescence in tumor than in normal tissue, and that the mean intensity was higher after administration of biparatopic 7D12-9G8-PS, compared to monovalent 7D12-PS. Intravital microscopy imaging however, revealed a heterogeneous fluorescence pattern in tumor that was different for the two conjugates. 7D12-9G8-PS showed a membrane bound fluorescence pattern in tumor, which was not observed for 7D12-PS. The reduced disassociation kinetics of 7D12-9G8-PS compared to 7D12-PS and/or the induced internalization of 7D12-9G8-PS might play a role in the observed higher fluorescence intensity and the more membrane bound fluorescence pattern for 7D12-9G8-PS 15. Previously, the highest fluorescence intensity of 7D12-PS was detected in tumor 30 minutes after administration with a clear decrease thereafter, whereas for 7D12-9G8-PS the fluorescence intensity remained high for at least 4 h after administration 16. 7D12-PS may be already dissociating from the receptor at early time points after administration and therefore showing less membrane localization at 1 and 2 h after administration. Internalization of 7D12-9G8-PS has been shown *in vitro* and was observed only once intravitally, possibly because of the many factors that influence the effective resolution of intravital imaging 24.

Consistent with the visual observation, colocalization analysis between GFP signal and photosensitizer fluorescence showed significantly higher Spearman's rank correlation coefficients, compared to the background, for both conjugates, suggesting tumor specific binding in time after administration which was significantly higher for 7D12-9G8-PS compared to 7D12-PS.While this study was focused on targeted PDT, targeted NB-PS conjugates have the potential to be used in fluorescence image guided surgery 51,61-63. Complete tumor resection relies on the surgeon's ability to differentiate between malignant and benign tissue but the infiltrative nature of cancerous tissue may hinder this. Fluorescence image guided surgery may assist a surgeon in delineating the cancerous tissue in the surgical field. NB-PS conjugates with their small size, high penetration and targeting characteristics, combined with fast clearance when unbound, could be excellent candidates for fluorescence image guided surgery. Clearance should be rapid enough to not have side effects of being photosensitive and long enough to last through the surgical procedure. Comparing the two conjugates in this study, 7D12-9G8-PS seems to be a good candidate, because of the high correlation coefficient between 7D12-9G8-PS fluorescence and tumor cells and the prolonged increased tumor fluorescence intensity in time after administration.

In normal tissue, a similar heterogeneous fluorescence distribution was found for both NB-PS, each showing fluorescence in the fascia and endothelial tissue up to 24 h after administration. We have previously detected NB-PS fluorescence in normal mouse tissues, such as tongue and skin, using fiber optic spectroscopy 16. But fiber optics spectroscopy reveals only information in intensity and not on cellular distribution. In the present intravital microscopy study, we observe that there are inhomogeneously distributed high intensities in the fascia and (close to) endothelial cells. Based on our current understanding of EGFR-targeted NB-PS, it is unclear why conjugates are present in normal tissue of the skin-fold chamber.

PDT induced damage is, in general, the result of a complex mixture of direct tumor cell damage, the influence of the (tumor) vascular responses and the immunological responses to these effects 1. In the previous study, we showed necrosis of the tumor combined with loss of CD31 and an increased presence of neutrophils after NB-PS mediated PDT 16. Our results here show membrane-localized fluorescence in tumor cells and also endothelial cell-associated fluorescence in the vasculature, thus considering the short diffusion distance of singlet oxygen, these are likely the primary sites of photodamage 27.

To assess the effects of NB-targeted PDT, first the morphology of the tumor cells was visualized using GFP fluorescence imaging. In some cases, the cellular GFP fluorescence pattern showed diffuse fluorescence and/or high fluorescence intensities confined to circular spots of different diameters, as early as 2 h after PDT, suggesting the degradation of tumor cells. In agreement with the difference in fluorescence intensity, and probably also with the differences in localization, more animals treated with 7D12-9G8-PS mediated PDT at 1 h post administration showed degradation of tumor cells compared to 7D12-PS. Based on these results, a difference in PDT-induced necrosis between the two conjugates might be expected but was not observed to be statistically significant. *Ex vivo* fluorescence imaging of HQ4, a necrosis avid cyanine probe 41,42, showed more tumor necrosis after 7D12-9G8-PS compared to 7D12-PS mediated PDT when light was applied 1 h after administration. However, while it is unknown if there is a linear relationship between HQ4 fluorescence intensity and necrosis, little can be said about the significance of this result. Histological evaluation of tumor necrosis showed slightly, but not significantly more, necrosis post 7D12-PS compared to 7D12-9G8-PS mediated PDT.

Vascular responses are a known contributor to the overall PDT response and those were broadly similar for both conjugates. Tumor vascular leakage was observed in more than 50% of the treated tumors within 2 h after PDT (Table 2). In our previous study we showed damage to the vasculature by histological evaluation 24 h post PDT 16. In the present study we did not find large differences in the magnitude of vascular responses for each conjugate. We did not investigate the effects of different NB-PS doses on the vasculature and overall response to PDT. It is likely that higher or lower doses of NB-PS will lead to different vascular responses in tumor and normal tissue depending also on the oxygen availability and the light applied.

The vascular effect of PDT could be utilized to enhance the EPR effect, in combined therapies with nanosized drug delivery systems 29-31. Employing a NB specifically designed to target the tumor vasculature could possibly increase this response even further. However, this should be investigated with caution, as tumor vascular stasis was also observed in 51-54% of the tumor areas 2 h after PDT, detected with rhodamine dextran fluorescence. This decreased perfusion was also confirmed through DCE-MRI of the subcutaneous tumor model, 1 h after 7D12-PS mediated PDT. This illustrates that any combination with PDT needs to be carefully investigated so that there is an optimal enhancement of the EPR effect. Tumor cell targeted, endothelial cell targeted, and conventional PDT might affect the vasculature differently. Therefore, intravital microscopic detailed studies, such as the one we present here, are critical to investigate a possible time window in which leakage could be exploited, before perfusion is compromised.

In line with the fluorescence localization of the conjugates, normal tissue showed significant changes in the vascular architecture of skin-fold chambers after PDT that were more severe for PDT at 1 h compared to 24 h. Histological examination revealed abundant damage to the subcutaneous musculature and fascia after PDT at 1 h but not at 24 h (Table 3). It would be interesting to investigate the vascular effects with a slightly longer time between administration and illumination, but significantly shorter than 24h, or with lower doses of the NB-PS conjugates. Perhaps then less vascular damage in normal surrounding tissues would be observed, though with the risk of decreased tumor damage, which after all is the main goal. Skin-fold chambers not transplanted with tumors showed similar changes in the vascular architecture (detected with intravital microscopy) but no damage to the musculature or to blood vessels (detected in tissue sections). This suggests that the vascular effects in normal tissue are transient when no tumor is present, and that, when tumor is present, the responding tumor contributes to the damage of surrounding normal tissue.

PDT at 24 h post administration was investigated in an attempt to utilize the higher tumor to normal ratio. Increasing the time between administration and illumination from 1 h to 24 h resulted in less vascular responses in tumor and normal tissue (much smaller tumor area showed lack of flow and less changes in normal vascular architecture) but also significantly less tumor response. At 24 h after administration, 7D12-9G8-PS still showed significant, although lower, colocalization of photosensitizer fluorescence with GFP suggesting that tumor cells would respond to PDT. However, the GFP fluorescence pattern of tumor cells after PDT had not changed, suggesting lack of tumor cell degradation. Indeed, histological evaluation showed a much lower necrotic tumor fraction after PDT at 24 h. In addition, HQ4 fluorescence also showed decreased necrosis in tumor and normal tissue after PDT at 24 h compared to 1 h. Although the TNR is much higher at 24 h after administration, the fluorescence intensity in the tumor was significantly lower. The current data clearly show that a high TNR is not the only factor in an effective treatment scheme: the concentration of NB-PS in the tumor is critically important. This result is in agreement with results from our previous study in the oral cavity 16.

The data presented here confirm that the overall acute response to EGFR-targeted NB-PS mediated PDT is not just a tumor cell specific response but a complex mixture of tumor cell responses and vascular effects. From a clinical perspective the combination of direct tumor cell death and tumor vascular damage is likely to be necessary for a maximally effective treatment.

In summary, we have obtained insights with unprecedented detail for targeted PDT, which are significant at present, since EGFR-targeted PDT using antibodies as carriers is currently being tested in phase I/II clinical trials 64. NB-PS conjugates 7D12-PS and 7D12-9G8-PS targeting EGFR showed significant tumor localization *in vivo,* which was higher for 7D12-9G8-PS. The significant higher tumor colocalization combined with the prolonged fluorescence intensity in time after administration makes 7D12-9G8-PS a good candidate for fluorescence image guided surgery. Illumination at 1 h after administration of either conjugates leads to significant tumor necrosis. Despite the difference in fluorescence intensity, no significant difference was observed in the overall acute tissue response; both EGFR-targeted NB-PS mediated PDT treatments lead to similar amount of tumor necrosis 2 days post treatment and no large differences in vascular responses. Growth delay studies are now needed to show the long-term efficacy of nanobody-targeted PDT.

## Figures and Tables

**Table 1:** Overview of nanobody-photosensitizer treatment schemes studied intravitally for distribution and tumor and normal tissue damage.

Group	n	Nanobody-PS conjugate	Illumination at	HQ4	Harvest after ill
1	8 *, **	7D12-PS	24 h	Yes	48 h
2	8 *, **	7D12-9G8-PS	24 h	Yes	48 h
3	3	Saline	24 h	Yes	48 h
4	8 *, **	7D12-PS	1 h	Yes	48 h
5	8 *, **	7D12-9G8-PS	1 h	Yes	48 h
6	3	Saline	1 h	No	48 h

* 1 animal without tumor, serving as normal tissue control. ** 1 animal sacrificed before PDT for fluorescence distribution.

**Table 2:** Number of animals showing vascular leakage in tumor and normal tissue 2 h post PDT using either 7D12-PS or 7D12-9G8-PS and illumination at either 1 or 24 h after administration.

Conjugate	Tumor		Normal tissue close to tumor	
	1 h	24 h	1 h	24 h
7D12-PS	4/6	2/6	1/3 *2	3/4 *1
7D12-9G8-PS	3/8	4/6	5/6	3/5

* number of animals not scored due to lack of flow

**Table 3:** Degree of tissue damage scored in H&E sections of the skin-fold chamber collected 48 h after PDT with 2 anti-EGFR nanobody-photosensitizer conjugates. Mean of 3 to 5 animals per group.

	Control	7D12-PS		7D12-9G8-PS		No tumor
		1 h	24 h	1 h	24 h	All NB-PS 1 or 24h
Damage to epithelium	-	+++	+	+++	-	-
Damage to muscle cells around tumor	-	+++	-	+++/++	-	-
Damage to muscle cells away from tumor	-	++	-	++/+	-	-
Damage to blood vessels around tumor	-	+++	-	+++	-	-
Damage to blood vessels away from tumor	-	++	-	++/+	-	-
Neutrophils	Few	Some	Some	Few	Few	-

## Abbreviations

PDT: photodynamic therapy
NB-PS: nanobody-photosensitizer
NB: nanobody
TNR: tumor to normal ratio
DCE-MRI: dynamic contrast enhanced magnetic resonance imaging
EGFR: epidermal growth factor receptor
ROI: regions of interest
OSC: oral squamous cell carcinoma
GFP: green fluorescent protein
AUC: area under the curve
H&E: hematoxylin and eosin staining
EPR: enhanced permeability and retention
