# Acute cellular and vascular responses to photodynamic therapy using EGFR-targeted nanobody-photosensitizer conjugates studied with intravital optical imaging and magnetic resonance imaging: Erratum

**DOI:** 10.7150/thno.93248

**Published:** 2024-01-09

**Authors:** Henriette S. de Bruijn, Vida Mashayekhi, Tom J.L. Schreurs, Pieter B.A.A. van Driel, Gustav J. Strijkers, Paul J. van Diest, Clemens W.G.M. Lowik, Ann L.B. Seynhaeve, Timo L.M. ten Hagen, Jeanine J. Prompers, Paul M.P. van Bergen en Henegouwen, Dominic J. Robinson, Sabrina Oliveira

**Affiliations:** 1 Center for Optical Diagnostics and Therapy, Dept. of Otolaryngology and Head & Neck Surgery, Erasmus MC Cancer Institute, Rotterdam, The Netherlands.; 2 Cell Biology Division, Dept. of Biology, Faculty of Science, Utrecht University, Utrecht, The Netherlands.; 3 Biomedical NMR, Biomedical Engineering, Eindhoven University of Technology, Eindhoven, The Netherlands.; 4 Division of Optical Molecular Imaging, Dept. of Radiology, Leiden University Medical Center, Leiden, The Netherlands.; 5 Amsterdam University Medical Centers, University of Amsterdam, Dept. of Biomedical Engineering and Physics, The Netherlands.; 6 Dept. of Pathology, University Medical Centre Utrecht, Utrecht, The Netherlands.; 7 Laboratory of Experimental Oncology, Dept. of Pathology, Erasmus MC, Rotterdam, The Netherlands.; 8 Pharmaceutics Division, Dept. of Pharmaceutical Sciences, Faculty of Science, Utrecht University, Utrecht, The Netherlands.

The authors regret to find an error in the published version of figure 1B, where the graph for 7D12-PS mistakenly was miscopied for 7D12-9G8-PS. During the review process a correct version of Figure 1B was included. The mistake was made while preparing the final text and figures in response to reviewers comments. The authors have revised Figure 1B, and confirm that the correction has no effect on the original data and conclusions. The authors apologize for any inconvenience that the errors may have caused.

## Figures and Tables

**Figure 1:**
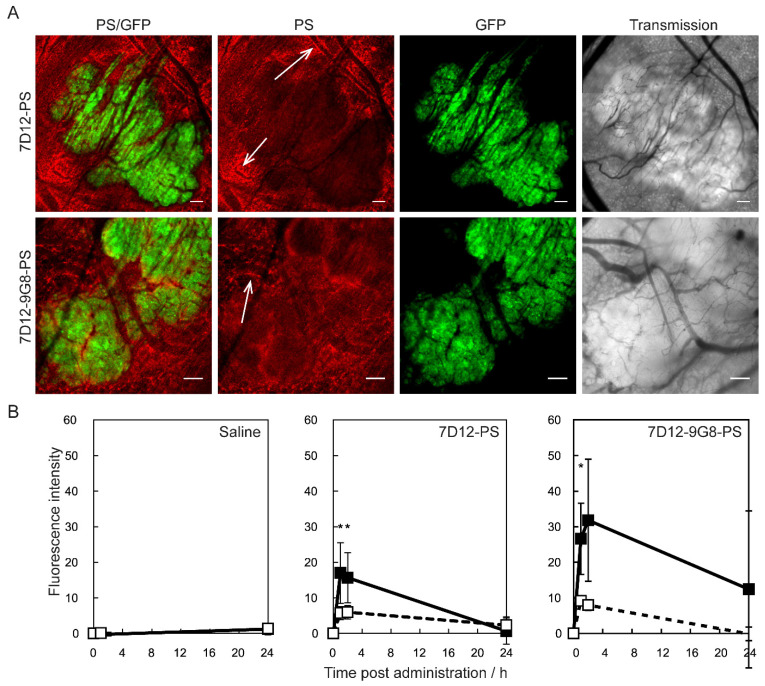
(A) Example of intravital fluorescence images recorded of the tumor in the skin-fold chamber 1 h after administration of 7D12-PS or 7D12-9G8-PS. Bar is 200 µm. White arrows highlight fluorescence close to vessels that surround tumor tissue. (B) Fluorescence intensity in tumor (solid squares and lines) and normal tissue far from tumor and not showing GFP signal (open squares and dashed lines) in the skin-fold chamber after administration of physiological saline, 7D12-PS or 7D12-9G8-PS. Weighted mean ± SD, n=3, 6, 8 respectively. Significant differences between tumor and normal tissue with p<0.05 (*).

